# Leveraging Open‐Source Geographic Databases to Enhance the Representation of Landscape Heterogeneity in Ecological Models

**DOI:** 10.1002/ece3.70402

**Published:** 2024-10-10

**Authors:** Tiziana A. Gelmi‐Candusso, Peter Rodriguez, Mason Fidino, Kim Rivera, Elizabeth W. Lehrer, Seth Magle, Marie‐Josée Fortin

**Affiliations:** ^1^ Ecology and Evolutionary Biology Department University of Toronto Toronto Ontario Canada; ^2^ Department of Conservation and Science Lincoln Park Zoo Chicago Illinois USA

**Keywords:** animal movement, confusion matrix, human disturbance, landscape configuration, mammal occupancy analysis, R programming, spatial ecology, urban wildlife ecology

## Abstract

Wildlife abundance and movement are strongly impacted by landscape heterogeneity, especially in cities which are among the world's most heterogeneous landscapes. Nonetheless, current global land cover maps, which are used as a basis for large‐scale spatial ecological modeling, represent urban areas as a single, homogeneous, class. This often requires urban ecologists to rely on geographic resources from local governments, which are not comparable between cities and are not available in underserved countries, limiting the spatial scale at which urban conservation issues can be tackled. The recent expansion of community‐based geographic databases, for example, OpenStreetMap (OSM), represents an opportunity for ecologists to generate large‐scale maps geared toward their specific research needs. However, computational differences in language and format, and the high diversity of information within, limit the access to these data. We provide a framework, using R, to extract geographic features from the OSM database, classify, and integrate them into global land cover maps. The framework includes an exhaustive list of OSM features describing urban and peri‐urban landscapes and is validated by quantifying the completeness of the OSM features characterized, and the accuracy of its final output in 34 cities in North America. We portray its application as the basis for generating landscape variables for ecological analysis by using the OSM‐enhanced map to generate an urbanization index, and subsequently analyze the spatial occupancy of six mammals throughout Chicago, Illinois, USA. The OSM features characterized had high completeness values for impervious land cover classes (50%–100%). The final output, the OSM‐enhance map, provided an 89% accurate representation of the landscape at 30m resolution. The OSM‐derived urbanization index outperformed other global spatial data layers in the spatial occupancy analysis and concurred with previously seen local response trends, whereby lagomorphs and squirrels responded positively to urbanization, while skunks, raccoons, opossums, and deer responded negatively. This study provides a roadmap for ecologists to leverage the fine resolution of open‐source geographic databases and apply it to spatial modeling by generating research‐specific landscape variables. As our occupancy results show, using context‐specific maps can improve modeling outputs and reduce uncertainty, especially when trying to understand anthropogenic impacts on wildlife populations.

## Graphical abstract

We leverage the resolution power of OpenStreetMap to improve the representation of landscapeheterogeneity in urban areas within LULC maps. To assess the information extracted with our framework, we conducted extensive testing in 34 North American cities, comparing our output to spatial data commonly used as landscape variables for ecological studies worldwide. The results demonstrated a ~90% accuracy in representing landscape heterogeneity, reinforcing the practicality and reliability of our approach. We included a case study to demonstrate the framework's applicability in ecological research where we developed a wildlife‐specific urbanization index representing habitat and shelter availability, vicinity to water resources, and road presence to analyze spatial occupancy of urban mammals. We predicted the spatial distribution of six mammal species across 93 camera trap sites in Chicago. We compared the predictions against using the “human influence index” as a landscape variable. The results from our case study demonstrated our framework strongly improved the model outputs, as it allows for higher resolution and context‐specific spatial variables.

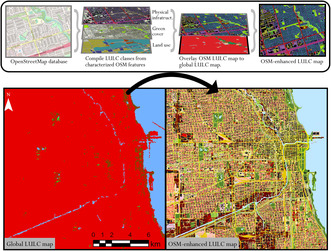

## 1 Introduction

Urban areas contain potential wildlife habitat composed of diversely managed green areas, which are embedded in a heterogeneous landscape of built environment segmented by linear features with varying types of usage and degrees of traffic. This landscape heterogeneity and its configuration can strongly influence wildlife spatial ecology, habitat use, and behavior (App et al. 2022; Hansen et al. 2020; Wurth, Ellington, and Gehrt 2020). Size, quality, and distribution of green areas can alter animal movement behavior (Smith et al. 2023), population dynamics (Huang et al. 2015; Vargová et al. 2023), and urban wildlife biodiversity (Aznarez et al. 2022; Strohbach, Lerman, and Warren 2013). Meanwhile, road network density can decrease habitat carrying capacity (Lyons et al. 2018), leading to indirect habitat loss through avoidance behavior (Dwinnell et al. 2019) and reduced species abundance (Rytwinski and Fahrig 2012). Therefore, it is important to carefully represent landscape heterogeneity when modeling ecological processes in urban areas.

Land cover and land use (LULC) maps characterize landscape heterogeneity and are used to describe the landscape in ecological models, generate environmental covariates for statistical models, and simulate wildlife adaptations to future landscape changes (Oyana, Johnson, and Wang 2014). On a global scale, LULC maps derived from satellite images enable wide‐scale spatio‐temporal comparisons of wildlife ecology. Unfortunately, most of the urban landscapes in global LULC maps are represented as a homogeneous “developed” or “built” class (ESA 2017; Karra et al. 2021). Such a coarse categorization of urban landscapes severely limits the applicability of these maps and hinders large‐scale inter‐city comparisons, which are necessary to further understand the effect of urbanization on biodiversity across spatial scales (Magle et al. 2019; Norton, Evans, and Warren 2016; Swan et al. 2021).

Given the spatial and thematic resolution limitation of global LULC maps within cities, most urban studies that analyze habitat use and wildlife behavior rely on LULC maps provided by local governmental agencies (Randa and Yunger 2006; Teitelbaum et al. 2020; Thompson, Malcolm, and Patterson 2021). These maps often have fine spatial resolution and urban‐specific landscape information such as land use classes, which provide a representation of human activity across the landscape, building footprints, and centerline layers representing road networks. Yet, LULC maps are sensitive to sampling bias derived from land cover classification, therefore local governmental maps might not be equivalent among cities, and therefore introduce bias to inter‐city comparisons (Thomlinson, Bolstad, and Cohen 1999). Furthermore, the availability of local governmental maps may be limited in countries with lower resources, excluding their representation in global analyses, such as countries in the global south (Zhang et al. 2023).

Remote sensing for urban land cover classification is making remarkable advances at the global scale using LiDAR (Light Detection and Ranging) and machine learning (Kuras et al. 2021). Finer‐scale representation of urban heterogeneity has been achieved using aerial photography and fine‐resolution satellite products (Cadenasso, Pickett, and Schwarz 2007; Pesaresi and Politis 2023a). However, these advances have high computational and economic costs, which limit their access to ecologists. Additionally, these data often have a limited representation of human usage patterns across the landscape—that is, land use—a key aspect of urban ecology (Gallo et al. 2022). Therefore, the need remains for a reproducible open‐source approach to generate a high‐resolution classification of urban landscapes, one that includes both the natural and human components.

Recent geographic advances have generated comprehensive open‐source databases, such as OpenStreetMap (OSM), that describe landscape features across the world, in fine detail. These databases represent an opportunity for ecologists to generate maps that depict landscape variables geared toward their research focus and could also be used to enhance the resolution of global LULC maps. OSM is an open‐source mapping platform storing landscape information (OpenStreetMap contributors 2017) in the form of geographic polygon features identified by a series of attributes. The database is generated by community members and supplemented with data from governmental and non‐governmental agencies around the globe. OSM has been reliably used in ecological research for extracting settlements and roads (Bide et al. 2023; Gizatullin and Alekseenko 2022), elevation (Shaykevich, Pašukonis, and O'Connell 2022), distance to water sources (de la Torre et al. 2021), and to study the fractal dimension of cities (Malishevsky 2022). Extracting a few features from the database for small areas can be done directly online. However, extracting a large study area, or extracting the full database to be able to characterize the landscape, requires understanding SQL language, the formatting particularities of the database storage files, and being able to identify the high number of features contained within. To increase the accessibility and applicability of such geographic databases to ecological studies, a roadmap is needed to illustrate the characterization, extraction, and processing of OSM features into landscape variables in a form that can be used in spatial analyses.

Hereby, we provide a roadmap to access the OSM database and apply its information to ecological analyses. First, we characterized OSM features fully describing urban landscapes, generating an exhaustive list of their respective attributes through an iterative manual process of identification and validation. Second, we designed a framework in R to generate maps derived from OSM features, where we extracted, classified, and processed the OSM features into raster‐based maps, and integrated them into a global LULC map, enhancing its informational resolution. We validated the framework by estimating the completeness and accuracy of its output, the OSM‐enhanced map, across the landscape. Third, we demonstrated the ecological applicability of an OSM‐enhanced LULC map by using it as an input to generate a wildlife‐specific urbanization index. The urbanization index we generated aimed at numerically representing habitat and shelter availability, human presence, and anthropogenic‐induced mortality risks. To evaluate the urbanization index we generated, we analyzed the spatial occupancy of six urban mammals in Chicago (Illinois, USA) and compared it against the human influence index (Wildlife Conservation Society—WCS and Center for International Earth Science Information Network—CIESIN—Columbia University 2005).

This study aims to increase the access of ecologists to open‐source geographic resources and facilitate fine‐scale spatial analyses in urban areas and global multi‐city ecological analyses. As such, along with our open‐access and reproducible R framework, we discuss potential ecological applications for the OSM database and improvement areas for the OSM database from an ecological perspective and make suggestions on how to overcome potential limitations when applying OSM to ecological studies.

## 2 Methods

### 2.1 Characterization of OSM Features Depicting the Urban Landscape

The OSM database consists of a series of geographic features represented as points (nodes), lines (ways), and polygons (relations; OpenStreetMap contributors, 2023). Each OSM feature is associated with a series of descriptive attributes (i.e., tags) consisting of key‐value pairs (e.g., land use: residential). Using the OSM dictionary and visualization tool, we characterized OSM features with attributes describing landscape components within urban areas and their surroundings (Tables S1 and S2). To obtain an exhaustive list of such features, we iteratively identified, extracted, and manually validated the completeness of the extracted features against aerial and street imagery, starting with one study area, and gradually increasing the number of study areas and countries sampled, manually characterizing features in overall 18 study areas, and ultimately validating the output generated using the characterized features across 34 study areas in the USA and Canada.

### 2.2 Step 1. Extraction of Features From the OSM Database

**FIGURE 1:**
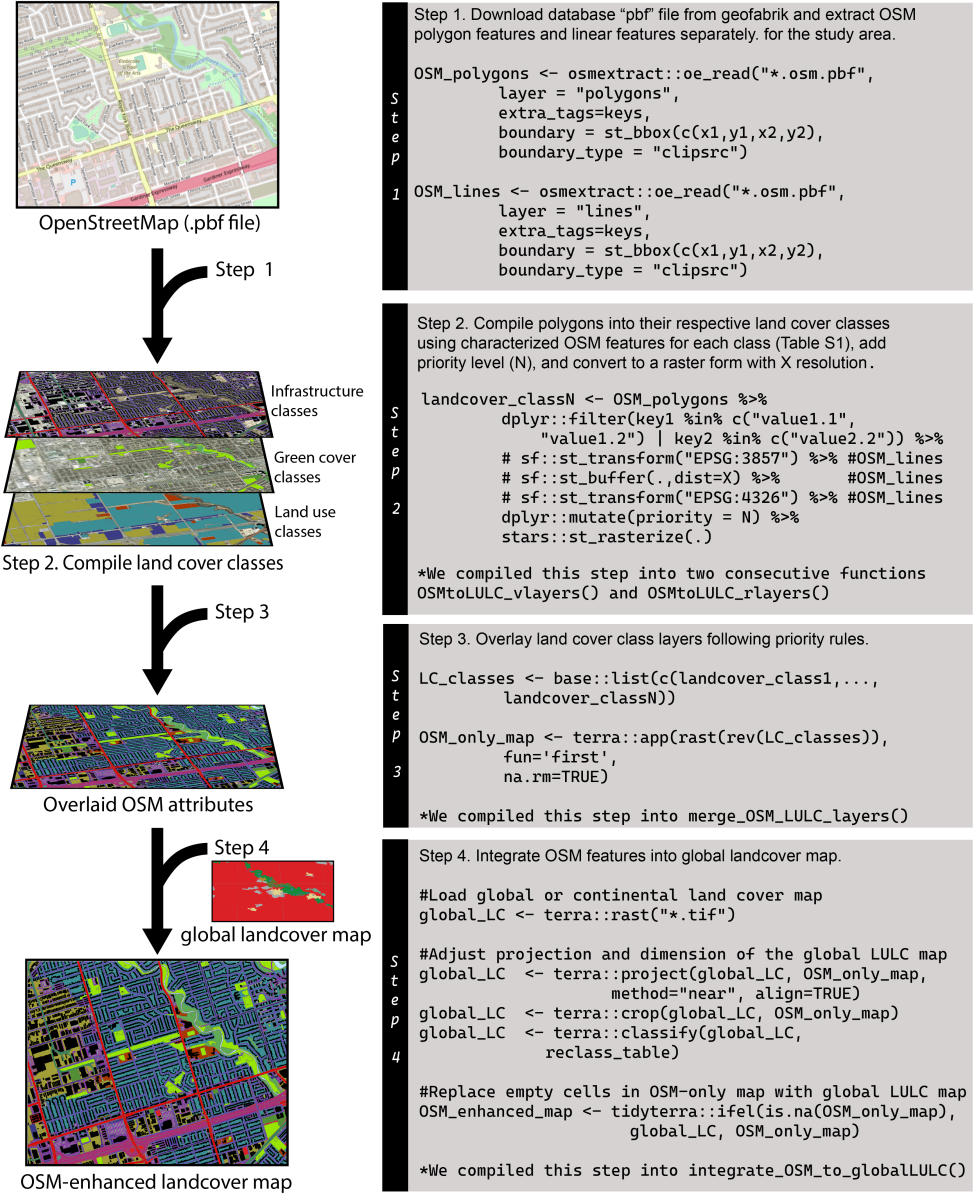
Visual representation of the framework for enhancing the resolution of developed areas in global landcover maps using OpenStreetMap (OSM) attributes. The representation follows the steps described in the methods section, along with representative R code for each step. In the R code representation, commented sections on theworkflow's script refer only to the layers containing linear features. Full code and functions are provided in the “OSM_for_Ecology” Github repository provided in the data availability statement.

We extracted the OSM features from the North American OSM database file (.pbf, *protocolbuffer binary format, geofrabrik* server) using the oe_read() function from the *osmextract* R package (Gilardi and Lovelace 2023; R Core Team 2023). The oe_get() function could be used instead to directly download and extract study areas with smaller OSM database files available in the geofabrik database. However, downloading the OSM database file directly from the geofabrik server has a faster and more reliable download process. The oe_read() function reads the features from the pbf database file and converts them into a simple feature collection of multipolygons. We did this separately for polygon features (layer = “multipolygons”) and line features (layer = “lines”) and for each, we extracted all the attributes of eachOSM feature (extra_tags = “keys”). To reduce computational strain, we extracted each study area separately by limiting the extraction tofeatures intersecting the study area's bounding box (boundary = sf::st_bbox(), boundary type = “clipsrc”) (Figure 1, Step 1).

### 2.3 Step 2. Classification of OSM Features Into Land Cover Classes

Once we extracted OSM features we organized them into land cover classes using the filter() function from the dplyr package (Wickham et al. 2023) compiling the previously characterized key‐value pairs into their respective land cover classes (Table S1). The land cover classification followed three main categories (human land use, green cover, and infrastructure), which were further subcategorized to represent urban landscape heterogeneity across 27 land cover classes. In detail, the three main categories (Tables S1 and S2) included: (i) human land use, encapsulating human activities classified by type (e.g., residential, commercial, industrial, institutional); (ii) green cover, classified based on vegetation density and management approach, as well as water cover and barren soil; and (iii) infrastructure, encompassing physical structures like buildings, fences, and linear features such as railways and roads classified by road type and hence their hypothetical traffic load. We converted linear features into polygons using buffers of varying sizes following linear feature types and observed mean lane numbers for roads (Table S3). While we extracted feature types to characterize urban intensity, other features could be queried and classified to meet other research goals. For example, a study focusing on the ecological implications of recreational areas may generate a landscape layer by filtering through OSM features using keys such as “leisure = dog_park” or “leisure = playground”. Further relevant keys and values can be found in Table S1 and using the OSM visualization tool by querying relevant geographic features on the map. After extracting the OSM features and classifying them, we converted each land cover class spatial feature layer into 30 m resolution raster‐based features using the rasterize() function from the terra package in R (Hijmans 2023). We opted for this resolution to reduce computational requirements given the substantial extent of our study areas, and to facilitate the integration of the OSM features into the global land cover map that also had a 30 m resolution. To reduce computational requirements for large study areas, the study area could be subdivided into smaller areas before rasterizing using the crop() function and merging back together using the mosaic() function, both from the terra package (Hijmans 2023). We compiled the code needed for this step into two functions, OSMtoLULC_vlayers() to filter the required OSM features in our framework and OSMtoLULC_rlayers() to convert the filtered OSM features into raster layers, one for each of the 27 classes (Figure 1).

### 2.4 Step 3. Processing of OSM Features Into an LULC Map

We overlaid the 27 classes of OSM features into a single LULC map by superimposing the raster layers following a priority structure (Table S1). In our case, we aimed to maintain potential animal movement paths and areas used as habitat to potentiate the use of our OSM‐enhanced output in spatial occupancy analyses and landscape connectivity assessments. As such, we overlaid human land use classes with green cover classes, and infrastructure classes were overlaid above all. Within the infrastructure layers, we overlaid buffered roads over buildings, roads were layered by road type (and characterizing traffic load), optimizing for overpasses and underpasses available to animal movement, and finally above all features, we overlaid hiking paths, railways, and fences, which generally run uninterrupted across the landscape, being potentially important features for animal movement within the city, acting as corridors or barriers (Gelmi‐Candusso et al. 2024; Kimmig et al. 2020). We compiled the code needed for this step into the function merge_OSM_LULC_layers() (Figure 1).

### 2.5 Step 4. Integration of OSM Features Into a Global Land Cover Map

To ensure no area was left uncharacterized, we integrated the compiled OSM features into a global land cover layer, whereby any cell devoid of OSM information was classified following the global land cover class. To enable such integration, we first reclassified the global LULC map into the 27‐class land cover classes described in our framework (Table S4) and introduced a new “developed unknown” class (the 28th class). We added this extra category to prevent misrepresenting human activity in the event OSM features were absent within the “built/developed” global land cover class (Figure 1 and Table S1). In this study, as a global LULC map, we opted for the CEC land cover data specific for North America (Commission for Environmental Cooperation (CEC) 2023), given our focus on North American cities. For intercontinental global applications, Copernicus (Buchhorn et al. 2020), or ESA WorldCover (Zanaga et al. 2021) maps are recommended. We compiled the code needed for this step into the function integrate_OSM_to_globalLULC() (Figure 1).

### 2.6 Validation of the OSM‐Enhanced LULC Map

We validated our framework's output in 34 study areas encompassing North American cities (Table S5). The study areas were defined by a squared envelope surrounding each city's administrative boundary with an arbitrarily defined buffer area ranging between 1 and 60 km (about 37.28 mi). We used a range of buffer sizes to include a diverse amount of peri‐urban and rural hinterland across study areas in our analysis (Table S5). The validation assessed two measures: completeness and accuracy.

We assessed the completeness of the OSM features included in our framework (Table S1) by investigating the number of OSM cells devoidof data after completing Step 3. To quantify the number of cells devoid of data, we estimated the absolute difference in total area between a map layer includingonly the extracted OSM features (output in Step 3) and the global LULC map layer. We did this for each land cover class separately. Furthermore, we assessed the completeness of our subcategorization system by quantifying the total area occupied by land cover classes with unknown subcategorization, whereby we compiled OSM features lacking the fine detail information required for their subcategorization (e.g., land use type, road type, and green cover type). Those classes respectively included the “developed unknown,” “roads unknown,” or “protected area” classes. To analyze the completeness results, we classified the cities based on proportion of urban landcover, for which we quantified the total surface area covered by the landcover classes in the Land use and Infrastructure categories (Table S2), divided by the total surface area of the study area.

The accuracy analysis of the OSM‐enhanced output consisted of cross‐validating 2123 sampling random points stratified by land cover class, against street view imagery and aerial photography (2023 Google). Across these samples, we quantified true and false positives and negatives. As accuracy measures, we estimated the Kappa statistic, which rates the agreement between two datasets given their agreement expected by random coincidence (Monserud and Leemans 1992), and the precision of each class, defining the rate at which each class correctly identifies the landscape, using the confusionMatrix() function from the caret package (Kuhn 2008). We focused on precision because we were interested in how often each landcover class we defined using the OSM database correctly characterized the landscape.

Furthermore, we investigated how extracting information from OSM compares to the information provided by commonly used remote‐sensing landscape characterization products in ecological studies. For this, we focused on the “buildings” class, an intrinsic infrastructure of urban areas that impacts wildlife movement and survival (Loss et al. 2014). Building footprints are the polygons delimiting each singular building on the land and can also be used to estimate building density and, along with roads, can be used to estimate impervious surface area, both landscape predictors commonly used in urban ecology (Haight et al. 2023). We compared the total building surface area obtained from the “buildings” land cover class in our framework's output against the total building surface area obtained from two remote sensing maps which are generally used to represent built surface areas globally: (1) a map including building information in terms of building density (GHL‐S, 10 m resolution, resampled to 30 m) (Pesaresi and Politis 2023b) and (2) a map including the building footprint (GHL‐MSZ, 10 m resolution, resampled to 30 m, Figure S4) (Pesaresi and Politis 2023a). As a reference for this comparison, we used local building footprint layers provided by local entities at the respective cities which were either generated computationally from fine‐resolution land cover layers, from aerial photography, or LiDAR (Light detection and ranging) (Table S6). We performed independent linear regressions between the reference values as response variables and the values of each of the other layers as fixed variables, (1) OSM, (2) GHL‐S, (3) GHL‐MSZ. We did these analyses for all study areas where a local building footprint layer was available (*n* = 20) while excluding cities where the local layers relied on the OSM database (Table S6).

### 2.7 Case Study: Spatial Occupancy Analysis

In this section, we assessed whether the OSM‐enhanced LULC map, which contains OSM features capturing urban landscape heterogeneity at a fine resolution (30 m), could be used to better estimate human disturbance of wildlife habitat in urban areas. For this, we used the OSM‐enhanced LULC map to generate an urbanization index. To assess how well the urbanization index can predict animal presence we used occupancy models to assess the relative fit between the urbanization index and the distribution of six urban‐adapted mammals. We re‐ran the analyses with the human influence index (Wildlife Conservation Society—WCS and Center for International Earth Science Information Network—CIESIN—Columbia University 2005) and compared the results for each species to those obtained with the urbanization index. The human influence index has a coarser resolution (1 km) and is commonly used for representing anthropogenic disturbance in large‐scale studies (de Oliveira et al. 2022; Di Pietro et al. 2023; Hill, DeVault, and Belant 2022; Martínez‐Gutiérrez et al. 2018; Sun et al. 2020).

### 2.8 Urbanization Index

The urbanization index was generated using the OSM‐enhanced land cover map as the data source, with the intention of representing fine‐scale human disturbance within cities, including habitat loss, human presence, road networks, and water availability. The urbanization index was created to be similar to the human influence index, whereby the landscape is scored following different components. In the case of the human influence index, these components include, among others, LULC maps, artificial light intensity and human population density (Venter et al. 2016). However, the urbanization index we generated was based solely on the OSM‐enhanced LULC map, considering vegetation land cover classes as representative of habitat availability, road networks and building footprints as representative of habitat loss and human presence, and the water land cover class as representative of water availability. With these land cover classes, we estimated (1) the surface area proportion of each vegetation land cover type in 900 m^2^ to include within developed areas the presence in terms of quantity and quality of surrounding vegetation at a scale relevant to animal movement between residential areas and surrounding green areas. We used the aggregate() function from the *terra* package with a factor of 30 and subsequently disaggregated to 450 m^2^ for overlaying purposes with the disagg() function from the terra package; (2) the proportion of building surface area in 450 m^2^, following the commonly used buffer radius size for occupancy models of 500 m (about 1640.42 ft); (3) a distance to the main roads layer (primary, secondary, and motorways) and railways; and (4) a distance to water layer. For the latter two, we used the distance() function from the *terra* package, these two map layers were in a 30 m^2^ resolution and the final values were standardized to a 0–1 range using each layer's minimum and maximum distance. We overlaid all components and generated the urban intensity metric through an arithmetic weighted sum within a 30 m cell‐size grid, where vegetation types were assigned different weights proportional to the amount of shelter provided for wildlife (3:2:1 ratio for forest, low‐height vegetation cover, and open green areas).

### 2.9 Camera Trap Survey

We collected animal presence and absence data using 93 motion‐triggered trail cameras (hereafter camera traps). The camera traps were deployed in urban greenspace from the center of downtown Chicago outwards along three 50‐km transects, with a minimum distance between cameras of 1 km. The sampling period included four sampling seasons within the year 2019, beginning in January, April, July, and October. The median number of weeks sampled across all seasons was 5 (minimum = 1 week, maximum = 7 weeks). Mammals detected within the images collected were classified by experts to the species level. These data were used to generate weekly detection histories for a suite of mammals commonly found in Northeastern American cities which included eastern cottontail (*Sylvilagus floridanus*), eastern gray squirrel (*Sciurus carolinensis*), raccoon (*Procyon lotor*), striped skunk (*Mephitis mephitis*), Virginia opossum (*Didelphis virginiana*), and white‐tailed deer (*Odocoileus virginianus*). For additional details on the sampling protocol, see Magle, Lehrer, and Fidino (2016); Magle et al. (2019). For reference, we calculated each species' average naïve occupancy across the four sampling seasons, which is the proportion of sites each species was detected at least once (Mackenzie et al. 2017).

### 2.10 Spatial Occupancy Analysis

As we collected data over multiple primary sampling periods, we used autologistic occupancy models, which are simplified dynamic occupancy models that use a first‐order autologistic term to account for temporal dependency in occupancy status between sampling seasons. The models were run using the auto_occ() function from the autoOcc R package v0.1.1 (Fidino 2024). To compare anthropogenic indices used as spatial covariates, we fitted three models to each species data and compared their relative fit with AIC (Burnham and Anderson 2004). We considered all models within 2ΔAIC of the top model to be competitive. The three models were, respectively, (1) a null model which included no spatial covariates, (2) a model including the OSM‐derived urbanization index as a spatial covariate, and (3) a model including the human influence index as a spatial covariate. All spatial covariates were summarized within 1 km of each camera trap site, and before the analysis, all values were centered and scaled.

## 3 Results

### 3.1 Landscape Heterogeneity Representation: Completeness and Accuracy

**FIGURE 2:**
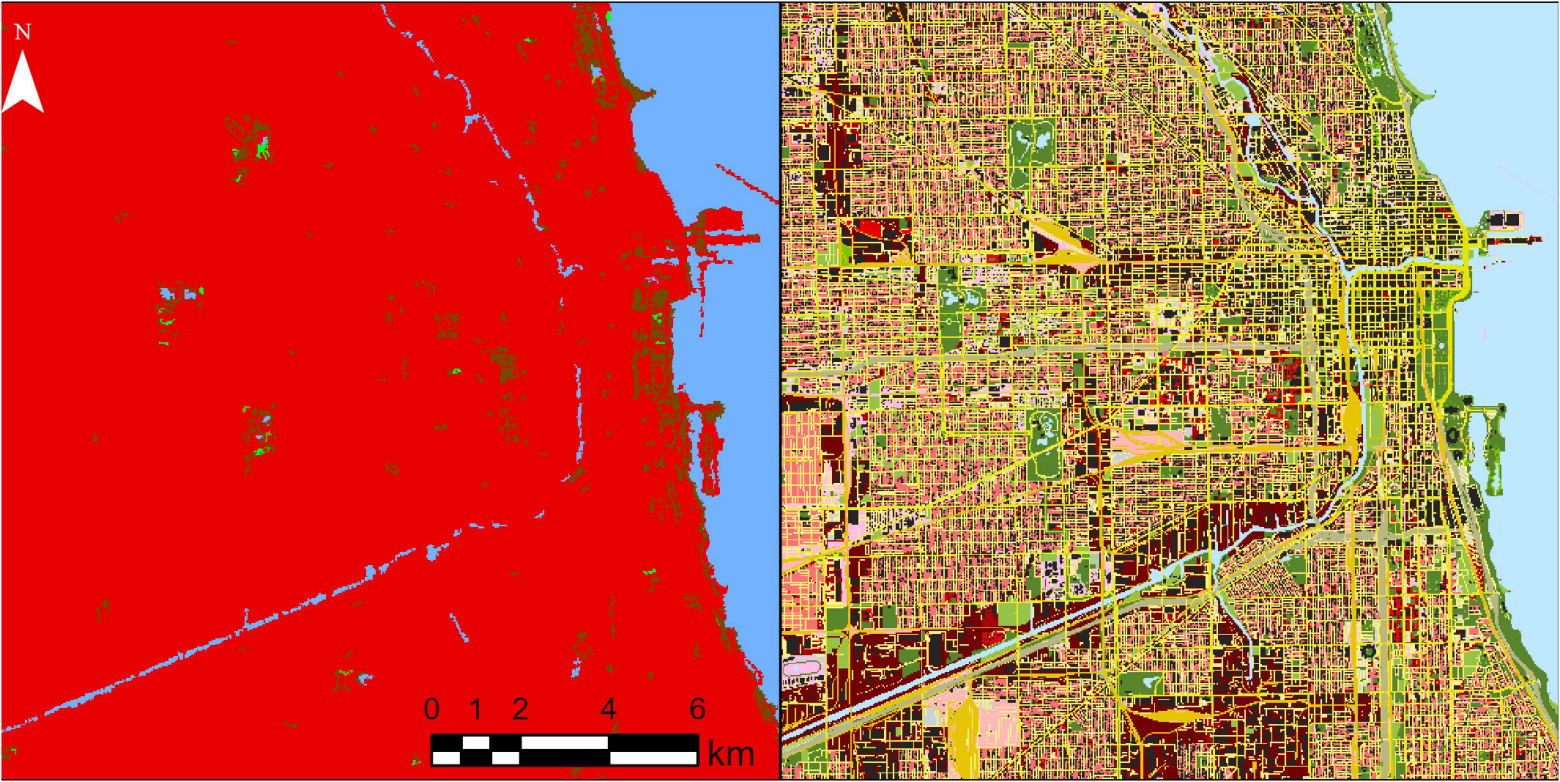
Visual comparison of the global land cover used in this study (Commission for Environmental Cooperation (CEC) LULC map, 30 m resolution, left), and the final output of our workflow, the OSM‐enhanced land cover map (30 m resolution, right) at the same location, in Chicago, Illinois, USA. Vegetation areas are represented in green shades, barren soil in brown, built environment or land use areas are represented in red shades, different road types are represented in yellow shades, and building footprints are represented in black.

Our framework enhanced the “developed/built” areas of the global land cover map by introducing urban landscape heterogeneity from varying human activities, building densities, and transportation networks within urban areas (Figure 2).

**FIGURE 3:**
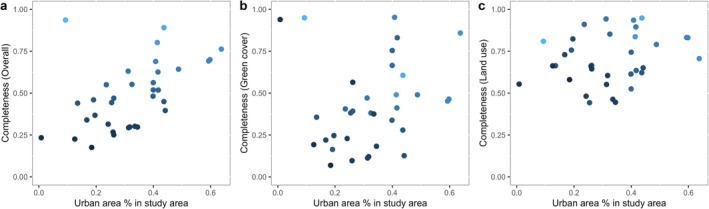
Completeness of the OSM features characterized in this framework, with the increasing proportion of the surface area occupied by developed land cover (i.e., land use and infrastructure) for 34 cities (dots, dark blue to light blue following increasing total completeness). (a) Overall completeness, including all 27 land cover classes, (b) completeness of green cover classes excluding water, and (c) completeness of impervious classes including land use (e.g., industrial, commercial, residential) and urban infrastructure (e.g. roads, buildings).

The final output of our OSM framework, where we combined OSM features and global LULC data, provided a complete and accurate representation of all land cover types within most cities. Overall completeness of the OSM features characterized, i.e. excluding any areas supplemented by the global LULC maps, increased linearly as the urban proportion of the study area increased (Figure 3a). This effect was predominant for green cover classes (Figure 3b), due to the low completeness these classes in hinterland non‐urban areas of some study areas with large buffer areas and some of the small rural cities analyzed, (Figure S1). In contrast, the completeness of OSM features describing impervious surface, i.e. “land use” and “infrastructure” classes, was higher (50%–100%) throughout all study areas, regardless of the proportion ofurban surface area included (Figure 3c). Completeness of OSM features describing waterbodies was low only for coastal study areas, as these included a large body of open water not represented in the OSM database (Figure S1). Furthermore, our completeness analysis showed that the percentage of areas occupied by classes with undefined sub‐categorization was low, meaning the number of OSM features that were missing the attribute information required for sub‐categorization was minimal (vegetation (class 7): 0.2%; roads (class 17): 1.6%, Figure S2).

In terms of accuracy, our framework's final output had a high Kappa coefficient of 0.89. In terms of precision rates (i.e., how often the description of the cell was correct), the classes with lower precision rates were water (class 12, Precision = 0.78), linear features in construction or abandoned (class 26, Precision = 0.56) and the developed/built class directly obtained from the continental scale land cover map when OSM information was absent (class 28, Precision = 0.35) (Table S7). The false positives in these classes included dried rivers, overgrown vegetation, and urban green cover classes, respectively (Figure S3a). When considering only classes with unclassified sub‐categorization, we found unclassified roads were predominantly residential (low traffic) or service roads (exceptionally low traffic), vegetation areas marked as protected were predominantly dense, heterogeneous, or open green cover, while unclassified developed areas were predominantly residential or some type of green cover (Figure S3b).

**FIGURE 4:**
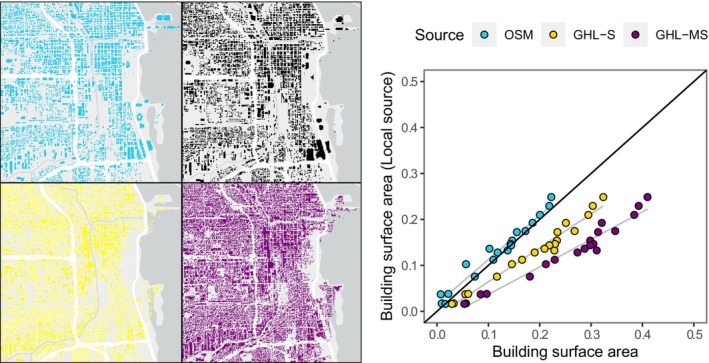
Left: Rasterized building footprints in Chicago, IL, USA, represented by the OSM building layer (OSM; cyan), remote sensing products, that is, Global Human Settlement Layer—Built‐up Surface (GHL‐S; yellow) and Global Human Settlement Layer—Morphological Settlement Zone (GHL‐MSZ; purple), and building footprints provided by the local institutions (City of Chicago, aerial photography, black). Right: Correlation between the total building surface area estimated from locally provided building footprints (*y*‐axis) and that estimated from the OSM‐enhanced LULC map (OSM; cyan) and the Global Human Settlement remote‐sensing Layers (GHL‐S; yellow and GHL‐MSZ; purple) (x‐axis).

Building surface area estimated from the OSM‐enhanced LULC map provided a closer estimate to those obtained with the building footprint provided by local governments and agencies. A linear regression between the values estimated from these two layers was close to 1 (*Y* = 0.02 + 0.96*x*, *p* < 0.001, *R* ^2^ = 0.96; Figure 4 and Table S8). In contrast, the two remote sensing products, GHL‐S and in particular GHL‐MSZ, significantly overestimated the total building surface area across cities when compared to our OSM‐derived output, negatively affecting the slope of the regression between estimated building surface area and building surface area from local sources (GHL‐S: *Y* = 0.01 + 0.74*x*, *p* < 0.001, *R* ^2^ = 0.97; GHL‐MSZ: *Y* = −0.02 + 0.60*x*, *p* < 0.001, *R* ^2^ = 0.96; Figure 4 and Table S8).

### 3.2 Spatial Occupancy Analysis

Striped skunks, white‐tailed deer, cottontail rabbits, Virginia opossums, raccoons, and Eastern gray squirrels were, respectively, detected on 140, 390, 401, 417, 600, and 895 camera trap weeks (Table S9). The average naïve occupancy across seasons for the previously listed mammals was, respectively, 0.17, 0.34, 0.43, 0.46, 0.55, and 0.72 (Table S10).

Models with the OSM‐derived urban intensity metric, the urbanization index, were the most competitive model for five of the six species (Table 1). Furthermore, species‐specific responses to the OSM‐enhanced urban intensity metric (30 m resolution) and human influence index (1 km resolution) were qualitatively similar, such that the direction of effect was the same for each species between models.

**TABLE 1:** The AIC results of the three autologistic occupancy models fitted to data of six urban adapted mammals collected via camera traps located throughout Chicago, Illinois, USA. The OSM‐enhanced model references the OpenStreetMap‐enhanced map used to generate the Urbanization index while the Human influence index (HFI) model used the human influence index data as a covariate. The null model had no spatial covariates. The best‐fit model is emboldened for each species.

Species	Model	ΔAIC	Cumulative weight
Cottontail rabbit	OSM‐enhanced	0.00	1
	Null	33.27	1
	HFI	34.15	1
Gray squirrel	OSM‐enhanced	0.00	1
	HFI	43.05	1
	Null	43.75	1
Raccoon	OSM‐enhanced	0.00	1
	HFI	25.89	1
	Null	34.26	1
Striped skunk	HFI	0.00	0.85
	OSM‐enhanced	4.38	0.95
	Null	5.67	1
Virginia opossum	OSM‐enhanced	0.00	0.75
	HFI	3.57	0.88
	Null	3.69	1.00
White‐tailed deer	OSM‐enhanced	0.00	1
	HFI	35.19	1
	Null	49.24	1

**FIGURE 5:**
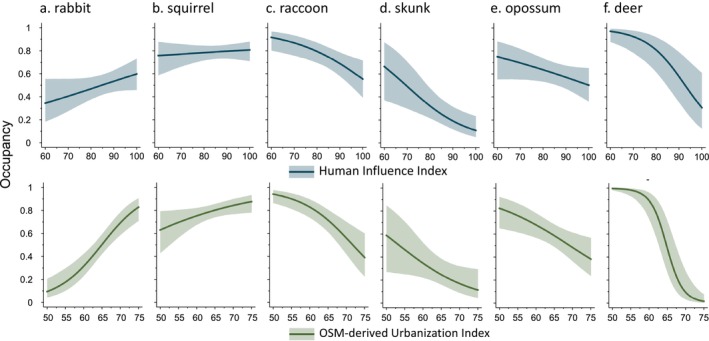
Occupancy response (0–1; y‐axis) of six mammal species cottontail rabbits (*Sylvilagus* spp.), eastern gray squirrels (*Sciurus carolinensis*), raccoons (*Procyon lotor*), striped skunks (*Mephitis mephitis*), Virginia opossums (*Didelphis virginiana*), and white‐tailed deer (*Odocoileus virginianus*) in the Chicago Area (USA), using the urbanization index and the human influence index as environmental variables (x‐axis).

Overall, species demonstrated both positive and negative associations with our OSM‐enhanced urban intensity metric. Specifically, we found a positive relationship between our OSM‐enhanced urbanization metric and the occupancy of cottontail rabbits (*β* = 0.67, 95% CI = 0.43, 0.95) and eastern gray squirrels (*β* = 0.27, 95% CI = 0.02, 0.52) and a negative relationship between this metric and the occupancy of raccoons (*β* = −0.54, 95% CI = −0.79, −0.28), striped skunk (*β* = −0.36, 95% CI = −0.70, −0.03), Virginia opossums (*β* = −0.34, 95% CI = −0.58, −0.09), and white‐tailed deer (*β* = −1.47, 95% CI = −1.96, −0.98). In our analysis, the OSM‐enhanced urban intensity metric at our camera trap sites ranged between 50 and 75, with larger numbers indicating more urban. When comparing the least to most urban locations sampled, cottontail rabbit occupancy increased by a factor of 8.8, and eastern gray squirrel occupancy increased by a factor of 1.2. Making the same comparisons, raccoon occupancy decreased by a factor of 2.4, striped skunk by a factor of 5.0, Virginia opossum by a factor of 2.15, and white‐tailed deer by a factor of 63.22 (Figure 5).

## 4 Discussion

The framework in this study, integrating the characterized OSM features into a global land cover layer, accurately represented urban landscape heterogeneity, which successfully translated into models with better explanatory power. Furthermore, the framework we detailed is highly generalizable across study areas and can be used to extract research‐specific landscape attributes and to generate new spatial metrics for analysis, as we did in our case study. Accuracy and flexibility make this a tool that will allow ecologists anywhere in the world to create rigorous landscape characterizations for their studies, especially as the OSM database continues its expansion.

Our validation results showed that OSM is the most accurate and complete within urban areas, particularly in the otherwise homogeneous developed/built global land cover class. Vegetation classes in the global LULC map we used were highly accurate (77.1%–86.9%), as are other global land cover maps following their own published accuracy rates (Buchhorn et al. 2020; ESA 2017), such as the ESA WorldCover (76.1%) or Copernicus global land cover map (80.6%). Therefore, as the proportion of non‐urban land increases in the study area using global land cover maps as a background to OSM features becomes more important. In addition, in these cases, it will be beneficial to include in the process further characterization of OSM features in the non‐urban landscape by querying and extracting features from the OSM visualization tool. For this manuscript we focused mainly on urban areas and their immediate surroundings, therefore we could say our keywords were characterized specifically for urban and peri‐urban areas so should not be taken for granted when analyzing predominantly rural study areas. Similarly, in areas where recent urban expansion has happened, it might be necessary to run a previous characterization of the areas where the land has recently changed. While OpenStreetMaps is updated daily, and we expect the database to expand as urban sprawl happens, the updating process depends on the community members' activity, and this might vary across cities. In contrast, given the high completeness in some cities of the OSM features proposed hereby (Figure 3c), some urban‐predominant study areas may be able to forgo the need to integrate the OSM features into a global LULC cover, stopping at step 3 of our framework. The quantification of void cells in the output of step 3, the OSM‐only map, will inform such a decision.

One interesting result from our analysis is that the OSM‐enhanced map had far more accurate building footprint data than standard remote sensing data products. While we expected buildings to be the most underrepresented class in the OSM database given the high effort required to add individual buildings into the OSM database, the total building surface area estimated from the OSM was closer to the area derived from building footprint products generated using Orthophotos and LiDAR, provided by local entities. As a result, we consider OSM building footprints alone can be a reliable source for estimates of fine‐resolution building density for cities where local governmental data is not available. Furthermore, if extracted in combination with road network data, building footprint can also provide estimates for overall impervious surface cover, which has been found to be a main driver of spatial ecological patterns in cities (Gallo et al. 2019; Haight et al. 2023; McCabe et al. 2018).

### 4.1 Case Study: Spatial Occupancy Analysis

Our case study showed that the OSM‐derived urbanization index better explained wildlife distributions across the urbanization gradient in Chicago for almost all species. Furthermore, when we compared the resulting species‐specific responses to urban intensity between our urbanization index and the human influence index, slope terms not only had the same directionality but agreed with the results of other analyses that used neither of these metrics (e.g., Haight et al. 2023). For example, cottontail rabbits and Eastern gray squirrels positively covaried with urban intensity and the remaining species negatively covaried with urban intensity. Given these comparable results among models using varying urban intensity metrics in the same study area, we conclude that even coarse urban intensity metrics can still capture a reduction in habitat availability as urbanization increases, at least when the sampling area includes the full urbanization gradient as it does in Chicago with 50 km (about 31.07 mi) long sampling transects. However, while habitat loss might be the main explanation for how species respond to urban intensity, we did find increasing spatial resolution greatly improved the explanatory power of the model, even in a large sampling area, noting the important contribution of fine‐scale movement decisions of urban mammals to overall occupancy patterns (App et al. 2022). Therefore, increasing the spatial resolution of landscape covariates will be most important for increasing the explanatory power of spatial occupancy models in study areas predominantly composed of high‐ and medium‐density urbanized land, as differences in habitat availability across sites become more subtle.

Besides resolution, another explanation for the lower explanatory power of the model using the human influence index might be the metric's limited scale when representing human presence within medium to high‐density urban areas, and in particular human presence as perceived by wildlife. Among other spatial products, this metric is derived from data on nighttime lights and residential population density. High upward light emission ranges within urban areas may decrease the sensitivity with which the metric explains human presence, limiting its variability within high‐density urban areas (Zheng et al. 2023). Furthermore, artificial lights might not fully represent human presence perceived as a threat by well‐adapted urban wildlife during nighttime, as terrestrial mammals with good movement capabilities might also be responsive to noise as a signal of imminent threat (Collins, Vickers, and Shilling 2022; Duarte et al. 2011; Willems, Phillips, and Francis 2022). Residential population density explains where the population resides but underestimates other areas of human activity such as commercial and industrial areas, which may also influence wildlife presence (Gelmi‐Candusso et al. 2024). In contrast, the numerical variability of our urbanization index mainly depends on the availability and typology of green areas and building footprint density, which our results suggest as a better representation of the landscape from a wildlife perspective, in terms of both human avoidance and resource/shelter availability. Overall, our case study concurs with previous research suggesting animal behavior is highly context‐dependent (Fahrig 2007; Owen, Swaisgood, and Blumstein 2017) and pinpoints how the availability of high‐resolution landscape data can be leveraged to generate context‐specific and research‐goal‐oriented representations of the landscape to improve ecological and statistical models.

### 4.2 Applications of OSM for Ecological Analyses

As seen in our case study, fine‐resolution landscape heterogeneity provides key information to better understand species distributions and has the potential to increase the explanatory power of spatial models. This is particularly useful for models predicting animal movement patterns (Braaker et al. 2014; Gelmi‐Candusso et al. 2024; Kimmig et al. 2020; Semeniuk et al. 2012; Watkins et al. 2015), habitat use (Thompson, Malcolm, and Patterson 2021), disease transmission (Dimitrov 2022), biodiversity patterns (Savage et al. 2015), and ecosystem services (Grafius et al. 2016). The availability of high‐resolution land cover maps also allows for the fine‐tuning of environmental variables, by generating better‐scaled metrics, such as the urbanization index we generated, the extraction of specific urban features relevant to a given research question, or simply in the form of a detailed land cover map. The framework we developed provides flexibility throughout its steps to allow for research‐specific questions and model‐specific applications. For example, in step 2, the key‐value pairs used to filter the OSM database can be modified to extract research‐specific landscape features by keeping only the keywords required in the parameters section of the filter() function, or by including different keywords queried from the OpenStreetMap Visualization tool. After features are filtered and extracted, a polygon layer is created, this can be used directly, omitting the land cover classification. Furthermore, the resolution at which the features are rasterized can be reduced (e.g., 10 m instead of 30 m) for more detail or increased (e.g., 100 m) to reduce computational requirements for modeling processes in large study areas. In step 3, the prioritization of the overlay of the raster layers can also be modified to better represent the landscape from a specific focus species perspective, for example, water class may be prioritized over linear features for research on aquatic wildlife. These modifications enable the fine‐tuning of the landscape characterization obtained from the OSM database toward the examination of different wildlife perspectives.

As substantial portions of the world only have access to coarsely classified global land cover data, our OSM‐enhanced workflow allows scientists to overcome geographic boundaries imposed by the availability of geospatial resources. Therefore, our framework can expand analyses to a global scale and allow for higher‐resolution comparisons across countries, especially in urban areas. As the world continues to urbanize at a staggering rate it is imperative to better understand how biodiversity responds to anthropogenic change. The workflow we described here helps achieve this, and as our case study shows, an OSM‐enhanced landscape characterization is a substantial improvement from the standard open‐access remote sensing products when representing the urban landscape, and in particular, when trying to understand how species respond to urbanization.

### 4.3 Potential OSM Improvements to Increase Its Applicability to Ecological Studies

When applying OSM to ecological studies it is important to consider three limitations and how they may be addressed. First, linear features contained incomplete and inconsistent information on lane numbers, which is crucial to buffer roads. To overcome this, we used a buffer of 6 m per lane and estimated the mean lane number for each road type, which our analysis shows is rarely missing within the database. Being able to buffer linear features systematically and accurately to a representative diameter could improve the applicability of OSM to animal movement and road mortality studies. Second, OSM data had a lack of information on vegetation types in private green spaces (e.g., residential yards). Most private green areas are classified as residential land use without further green cover classification. To better represent residential green cover, given the fine resolution and predominantly high completeness within urban areas, a logical assumption would be to consider the areas not overlapped by buildings, parking lots, or linear features as residential green areas. To incorporate a measure of vegetation density representing resource and shelter availability, these areas may then be overlaid to a vegetation index (e.g., Pesaresi and Politis 2023a). A particularly important step when applying the framework to study subjects strongly affected by yard vegetation complexity, for example, hedgehogs (App et al. 2022). Third, land use information was sparse in certain study areas, reaching up to 20% of a total study area where information on human activity was absent (Figure 2c). This might be problematic for ecological studies focusing on animal species with a high degree of human avoidance, which might differentially favor land use classes, for example, coyotes favoring residential areas during specific times of day and specific social status (Thompson, Malcolm, and Patterson 2021). To overcome this, community efforts should be directed toward completing land use information in the OSM database, see Table S5 for completeness values in the cities analyzed here. Our analysis in study areas where OSM land use information was scarce shows those unclassified land use areas were predominantly residential. However, before making assumptions for landscape reclassification and model parametrization, scientists should locally verify the percentage of missed land use area by quantifying the proportion of the study area covered by the unclassified developed class (class 28), and, if available, examine true land cover through online aerial and street imagery.

## 5 Conclusion

The framework we detailed can increase the resolution of global urban land cover maps and be used to generate research‐specific environmental variables for ecological studies. The OSM‐enhanced landscape characterization generated by our framework is an improvement from other maps that represent urban areas, primarily in terms of accuracy, accessibility, and geographic scale of applicability. We unravel limitations, potential solutions, and considerations for implementing the OSM database into ecological studies. Furthermore, the proposed urbanization index, derived from the OSM‐enhanced land cover map, is an example of how fine‐resolution information can be leveraged to generate context‐specific landscape representations that may increase the explanatory power of statisticalmodels. Studies benefiting from integrating a fine representation of landscape heterogeneity include modeling approaches investigatinganimal spatial distribution and movement, epidemiological analyses, and landscape connectivity. Rather than relying on maps generated by local governments, by leveraging the global‐scale OSM database we can enable worldwide multi‐city comparisons and allow for the inclusion of critically underrepresented regions.

## Author Contributions

**Tiziana A. Gelmi‐Candusso:** conceptualization (equal), formal analysis (equal), funding acquisition (supporting), methodology (equal), software (equal), validation (equal), visualization (lead), writing – original draft (lead), writing – review and editing (lead). **Peter Rodriguez:** conceptualization (equal), methodology (equal), software (equal), validation (equal). **Mason Fidino:** data curation (equal), formal analysis (equal), visualization (equal), writing – review and editing (equal). **Kim Rivera:** data curation (equal), writing – review and editing (equal). **Elizabeth W. Lehrer:** data curation (equal), writing – review and editing (equal). **Seth Magle:** funding acquisition (equal), writing – review and editing (equal). **Marie‐Josée Fortin:** funding acquisition (lead), writing – review and editing (equal).

## Conflicts of Interest

The authors declare no conflicts of interest.

## Supporting information


Data S1.

## Data Availability

Data is available directly from the OpenStreetMap database: https://www.openstreetmap.org/export, and R script for our framework is available at the corresponding author's GitHub repository: https://github.com/tgelmi‐candusso/OSM_for_Ecology.
